# Effects of a Tai Chi-Based Stroke Rehabilitation Program on Symptom Clusters, Physical and Cognitive Functions, and Quality of Life: A Randomized Feasibility Study

**DOI:** 10.3390/ijerph18105453

**Published:** 2021-05-20

**Authors:** Rhayun Song, Moonkyoung Park, Taejeong Jang, Jiwon Oh, Min Kyun Sohn

**Affiliations:** 1 College of Nursing, Chungnam National University, Daejeon 35015, Korea; songry@cnu.ac.kr (R.S.); ohjw@cnu.ac.kr (J.O.); 2 College of Nursing, Woosuk University, Wanju 55338, Korea; wwtbi@woosuk.ac.kr; 3 College of Medicine, Chungnam National University, Daejeon 35015, Korea; mksohn@cnu.ac.kr

**Keywords:** stroke, Tai Chi, symptom cluster, physical functional performance, quality of life

## Abstract

Stroke survivors suffer from disease-associated symptoms. Tai Chi can be a beneficial approach to provide an adapted form of intervention to manage their symptoms. The study aimed to determine the effects of a Tai Chi-based stroke rehabilitation program on symptom clusters, physical and cognitive functions, and stroke-specific quality of life among stroke survivors in Korea. Thirty-four stroke survivors were randomly assigned to receive either the Tai Chi-based program or the stroke-symptom management program. The feasibility of the program and its effects on the outcomes were assessed at baseline, 3 months, and 6 months. Repeated measures ANOVA showed that most symptoms improved in both groups during the 6-month period, but swallowing-related symptoms improved significantly in the Tai Chi group. Based on the interaction effect, Tai Chi was more effective on flexor muscle strength, ambulation, and activities of daily living and cognitive function over 6 months than their counterparts. Among SS-QOL dimensions, the Tai Chi group showed significant improvements in the thinking and self-care dimensions. The Tai Chi-based stroke rehabilitation program was feasible and safely applicable to stroke survivors in the community settings. This program could improve symptoms, physical and cognitive function, leading to improvements in the self-care dimension of the SS-QOL among stroke survivors.

## 1. Introduction

Cerebrovascular disease is a major global concern and individuals with stroke suffer from disease-associated symptoms that influence their physical and cognitive functions in everyday life [1,2]. Due to hemiplegia, which is the most devasting and long-lasting symptom, stroke survivors experience physical difficulties with speaking, balancing, coordinating, walking, and vision, and those problems can cause depression and a poor quality of life (QOL) [1]. The symptoms vary according to the affected site and size in the brain of the initial stroke lesion, which makes the recovery processes more complicated and individually different [3]. Therefore, approaches involving multidisciplinary teams are crucial for the development of effective stroke rehabilitation programs that can address various needs regarding the physical, social, cognitive, and psychological functions of stroke survivors [3]. 

Tai Chi is a low-intensity aerobic exercise method originated from an ancient Chinese martial art and characterized by the fundamental principles of slow, smooth, and continuous body movements that involve transferring the body weight while maintaining a relaxed upright posture [4]. It is also well known for its psychological benefits deriving from the mind–body connection rooted in Taoism [4,5]. These characteristics of Tai Chi have led to it being widely practised, not only for improving physical problems (e.g., postural balance, movement coordination, muscle strength, and flexibility) but also for calming the mind and meditating [5,6,7,8]. Many studies have found that performing Tai Chi improves balance and gait, decreases the incidence of falls [9,10], and relieves depression, anxiety, and stress [11,12]. 

Considering the functional limitations of stroke survivors, safety should be the main concern when applying any form of exercise to this population. Adapted forms of Tai Chi have been applied to populations with poor standing balance, such as stroke patients [13], older adults using wheelchairs [14], and individuals with spinal cord disorder [15]. Previous feasibility studies have found that Tai Chi can be safely and effectively applied to those with restricted functioning in an adapted form while either standing or sitting. A study that applied Yang-style Tai Chi as a community-based physical activity program to stroke survivors found that 28 subjects with a mean age of 69 years were able to complete Tai Chi movements three times a week over a 3-month period with an adherence rate of 92%, and no occurrence of adverse events [16]. Wayne et al. [17] similarly reported the positive effects of Tai Chi applied to elderly subjects with impaired cognitive function. A randomized trial examining the effects of seated Tai Chi in 60 long-term-care residents found significant improvements in their QOL and depression scores after they performed seated Tai Chi three times a week for 26 weeks [14]. 

For stroke survivors who usually find it more challenging to access or utilize rehabilitation programs after discharge, Tai Chi can be a beneficial approach that is consistent with their diverse abilities [8,18,19,20]. However, explorations of the feasibility of implementing Tai Chi as a stroke rehabilitation program based on the outcomes of stroke-related symptoms and physical and cognitive functions of stroke survivors are still in a very early stage of investigation. Therefore, this study applied a Tai Chi-based stroke rehabilitation program to stroke survivors at various rehabilitation stages for 6 months to determine the program effects on stroke-symptom clusters, physical, psychological and cognitive functions, and QOL. 

## 2. Materials and Methods

### 2.1. Study Design 

A single-blind randomized controlled trial was conducted to compare the effects of the 6-month Tai Chi-based stroke rehabilitation program with a stroke-specific symptom management program (ClinicalTrials.gov identifier: NCT028688 40). The data collection process needed about 8 months to recruit 34 stroke survivors who agreed to participate in the study, and consequently the study period was around 15 months (from February 2016 to May 2017), which included 6 months to complete the Tai Chi or symptom management program. 

### 2.2. Participants

Stroke survivors registered at the outpatient rehabilitation center were screened by their primary physician applying the inclusion criteria of being: (1) diagnosed with stroke, (2) referred by a physician to the rehabilitation therapeutic program and (3) who have a mobile phone and can use the text functions, and the exclusion criteria of (1) not being able to understand questionnaires or (2) not being able to stand unaided during functional tests. The research team explained the purpose of the study to the potential candidates, who then signed a consent form. After the subjects completed the baseline measures, they were randomly assigned either to the Tai Chi or control group. This study was approved by the institutional review board (IRB approval number: 2-1046881-A-N-01-201505-HR-020) where the primary investigator was affiliated. 

### 2.3. Sample Size

**Figure 1:**
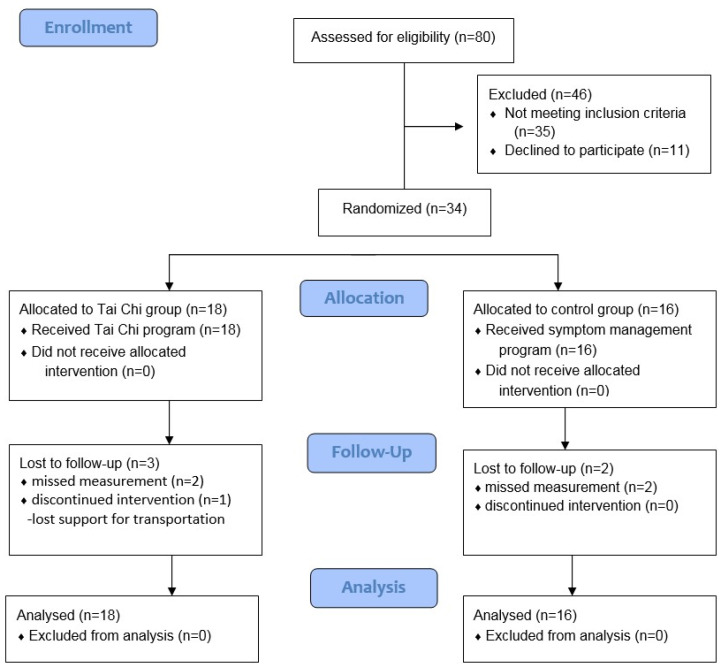
Flow diagram of the study.

The required sample size was determined for the present study based on a previous study [14] for a reported effect size of 0.89 (mood), a power of 0.8, and two-tailed independent group differences. It was found that 40 stroke survivors were required, and 34 participants (18 in the Tai Chi group and 16 in the control group) were recruited by applying the inclusion criteria during the 8-month recruitment process, with dropout rates of 16.6% and 12.5%, respectively. Using the expectation-maximization method for imputing missing data, all 34 subjects were included in the analysis (Figure 1).

### 2.4. Randomization and Masking

Random numbers obtained from www.randomizer.org were used to assign subjects to the Tai Chi and control groups. One researcher who was not involved in patient recruitment performed the random assignment when a new stroke patient agreed to participate in the study, and also completed the pretest measures. The participants were notified about their group assignment 1 week prior to their first class of the program. 

### 2.5. Treatments

An adapted form of Tai Chi for health programs—which shares the common Tai Chi principles but was modified for those with restricted mobility—was used as the exercise component of the stroke rehabilitation program for stroke survivors. The Tai Chi class as a group was led by a trained Tai Chi instructor twice a week for 6 months in the physical therapy room of the university hospital rehabilitation center. Each session consisted of a 5-min warm up, a 5-min of qigong, a 35-min of Tai Chi movements in a seated or standing position, and a 5-min cool-down. Chairs were available for safety and comfort while patients were performing seated Tai Chi. Any signs of discomfort were closely monitored by a research assistant and a rehabilitation center staff during each session. Various motivation strategies were applied in an effort to reduce the dropout rate, such as promoting the goal of performing self-exercises regularly at home, providing Tai Chi shirts when attending at least 80% of the sessions over 3 months, and making weekly follow-up calls to remind the subjects about performing home exercises and the next schedule of the program. 

During the study period, 16 participants in the control group received the symptom management program. A research assistant assigned to the control group provided weekly text messages with information about stroke symptoms at different rehabilitation stages and how to manage those symptoms, as well as the contact number to call or send a text message to when they had any questions about symptom management. Due to the difference in modality provided, there was no episode that the participants have come in contact with each other between the experimental and the control groups.

### 2.6. Feasibility Monitoring

The intensity of the intervention was low at first, allowing the participants to be familiarized with the movements, and progressively increased within their comfort zone. The rate and steps to increase the dose of the program were recorded to make the standardized protocol in Tai Chi for stroke rehabilitation. At the end of the intervention, exit interview was conducted with five participants who agreed to explore their experience in the program.

### 2.7. Measures

The outcomes were measured in both groups at the baseline, at 3 months, and at 6 months. A standardized questionnaire for obtaining information about demographic characteristics, cognitive function, stroke-specific QOL, and symptom clusters was administered via face-to-face interviews by trained research assistants. The outcomes for physical function and muscle strength were measured at the rehabilitation clinic by specialized technicians. All outcome assessors were blind to the group assignments.

#### 2.7.1. Cognitive Function

Cognitive function was measured using the Korean version of the Montreal Cognitive Assessment (K-MOCA) [21] and the Korean version of the Mini Mental State Examination (K-MMSE) [22]. The K-MOCA assessed cognitive function in the areas of visuospatial function, executive function, naming, memory, attention, language, abstraction, and delayed recall. The K-MOCA reportedly has criterion validity with the K-MMSE (r = 0.65), and Cronbach’s α values of 0.81 to 0.84.21.

#### 2.7.2. Muscle Strength

Knee muscle strength was assessed by isokinetic muscle testing using the System 4 Pro system (Biodex Medical, Shirley, NY, USA) to measure knee flexion and extension peak torque of both the involved and healthy legs. 

#### 2.7.3. Balance

Balance was measured using the Berg Balance Scale (BBS) [23,24] The BBS is a 14-item scale that quantitatively assesses balance through direct observations of the performance ability, with each item scored from 0 (‘inability to complete the task’) to 4 (‘independent completion’). The 17-item Trunk Impairment Scale (TIS) [25] was used to assess upper-body balance and coordination, with 3 items for static balance, 10 items for dynamic balance, and 4 items for coordination. The test–retest reliability of the TIS has been reported (r = 0.87 to 0.96), as has its good interrater reliability (r = 0.85 to 0.99).

#### 2.7.4. Ambulation and Motor Function

The Functional Ambulation Category [26] was used to assessed the ambulation ability of stroke patients on a 6-point scale from 0 (‘unable to walk’) to 5 (‘can walk freely), based on how much human support was required to walk. The modified Rankin Scale (MRS) was also used to assess recovery of motor function, with scores from 0 (‘no symptoms at all’) to 5 (‘severe disability’) and 6 (‘dead’). The MRS showed good test–retest reliability (kappa = 0.81 to 0.95), and its construct validity with neurological impairment and its convergent validity with another disability scale have also been documented [27]. The Korean version of the Modified Barthel Index (K-MBI) measures the performance of the activities of daily living (ADL), and is a reliable and valid measurement for assessing the functional status of stroke patients [28].

#### 2.7.5. Stroke-Specific Quality of Life

The perceived QOL was measured using the Korean version of the Stroke-Specific Quality of Life questionnaire (SS-QOL), which evaluates the level of energy, family roles, language, mobility, mood, social roles, personality, thinking, and self-care using a 5-point Likert scale. The validity of psychometrics measures of the original version of the scale was reported, with a reliability coefficient of 0.73–0.89 [29]. Cronbach’s α values for the subscales of the SS-QOL were 0.92–0.93 among stroke survivors [30]. 

#### 2.7.6. Stroke-Symptom Clusters

Stroke-related symptoms were measured using the Korean version of the Stroke Symptom Cluster Scale (SSCS-K) [30]. The SSCS-K has 65 items in the six subscales of mobility, sensory, cognition, communication, mood, and swallowing. The psychometrics of SSCS-K was described in the original study involving a Korean stroke population [30], and demonstrated acceptable reliability (Cronbach’s α = 0.92), with Cronbach’s α ranging from 0.91 for cognition to 0.92 for mobility.

### 2.8. Statistical Analysis

Data were analyzed using SPSSWIN (version 20.0) software (IBM Corp, Armonk, NY, USA). Descriptive statistics were applied to the demographic and stroke-related characteristics of the participants. Independent *t*-tests were used to assess homogeneity between groups for outcome variables in the baseline measurements. Repeated-measures ANOVA was used to analyze time, group, and interaction effects of the program. The significance threshold (alpha) was set as 0.05.

## 3. Results

### 3.1. Participant Characteristics

The mean age of the subjects was 57.9 years, and their duration of stroke was 9.16 ± 7.36 months. Most of the participants were married (76.5%), not currently employed (79.4%), and had a low socioeconomic status (57.6%). Most of them (70.6%) were able to perform the ADL independently. No significant group differences were found in demographic and stroke-related characteristics (Table 1). 

### 3.2. Homogeneity Tests at Baseline 

Group comparisons at the pretest revealed no significant differences in the outcome variables between the groups (Table 2). The mean attendance rate for the study participants was 83.5% during the 6-month program.

### 3.3. Treatment Effects on Stroke-Symptom Clusters

At the completion of the 6-month Tai Chi-based stroke rehabilitation program, both groups showed improvements in stroke-related symptoms specifically in the areas of movement, cognition, and communication (Table 3). 

The only significant intergroup difference in symptom clusters was for swallowing difficulties. The Tai Chi group showed significant improvements in swallowing-related symptoms over 6 months compared with control group (interaction effect: *F* = 8.96, *p* = 0.001).

### 3.4. Treatment Effects on Muscle Strength and Physical and Cognitive Functions

The Tai Chi group showed significant improvements in muscle strength, specifically for flexor muscles on the involved side (interaction effect: *F* = 8.61, *p* = 0.002). Both groups showed significant improvements in balance as measured using the BBS and TIS over time, but no significant interaction effects were found. Mobility and the ADL improved in both groups over time, but the significant improvement existed in the Tai Chi group (Interaction effect: *F* = 6.78, *p* = 0.002 for mobility; *F* = 3.23, *p* = 0.046 for ADL). Cognitive function also improved significantly in the Tai Chi group (Interaction effect: *F* = 7.09, *p* = 0.004 for K-MOCA; *F* = 4.33, *p* = 0.017 for K-MMSE) (Table 4). 

### 3.5. Treatment Effects on SS-QOL

At the completion of the 6-month Tai Chi-based stroke rehabilitation program, the Tai Chi group showed significant improvements in some dimensions of the SS-QOL, specifically in thinking (interaction effect: *F* = 3.70, *p* = 0.030) and self-care (interaction effect: *F* = 8.82, *p* = 0.001). No significant interaction effects were found in the other dimensions of the QOL (Table 5).

## 4. Discussion

This study aimed to determine the feasibility and effectiveness of Tai Chi-based stroke rehabilitation among a group of stroke survivors with a mean age of 57.9 years at various rehabilitation stages ranging from 3 to 24 months. During the exit interview at the end of the program, the participants expressed they were able to follow the movements seated or standing, while enjoying being in the supportive group. The program was safely applied to stroke survivors for 6 months, and no falls or other adverse effects related to the intervention were reported by the participants or research staff during the study period. Performing Tai Chi while either seated or standing for 100 min per week was well tolerated as progressed within their comfort zone. The mean attendance rate was 83.5% for those in the Tai Chi group, and the dropout rate was 16.6% in the Tai Chi group and 12.5% in the control group. The main reasons for the dropouts were unrelated to the intervention, including missed measurements (n = 3) and discontinuing the intervention due to lost support for transportation (n = 1).

The study found that the Tai Chi-based stroke rehabilitation program was partially beneficial to stroke survivors in terms of physical and cognitive functions, and some dimensions of the QOL. While the stroke-related symptoms of the patients in both groups improved during the 6-month study period, those in the Tai Chi group showed significant improvements in swallowing difficulties over time compared with the controls. The comparison of stroke-symptom clusters across the rehabilitation stages showed that swallowing difficulties remain priority symptoms that most stroke survivors experienced for up to 5 years [30]. Swallowing difficulties are known to be one of the serious barriers hindering the path to recovery for stroke survivors, which significantly affects their QOL [31]. The mechanisms of improving swallowing ability through Tai chi are not clear, but may be explained by two assumptions. First, mindful eating is emphasized through Tai Chi practice that helps the participants to take enough time to eat with better sensual awareness of food, consequently may reduce the risk of aspiration. Second, head rising excises such as Shaker excise can improve swallowing by enhancing the functionality of the upper esophageal spinster muscle through isotonic and isometric excise [32]. The warm up exercise for Tai Chi exercises include neck flexion and extension exercise, which may have strengthened the muscles around the neck and helped improve swallowing. Yet, the underlying mechanisms need to be confirmed in further studies. 

A focused review on Tai Chi for stroke rehabilitation analyzed four intervention studies, with the results suggesting that Tai Chi is beneficial to improving physical and social functions of the QOL and mental health among stroke survivors [33]. We similarly found that the participants in the Tai Chi group reported significant improvements in the thought-processes and self-care dimensions of the QOL when assessed using the SS-QOL. The specific dimensions of the SS-QOL were strongly associated with corresponding functionality measures [34], which was also supported by our study findings for physical- and cognitive-function outcomes.

Since the mobility problems improved over time, our findings support the hypothesis that even an adapted form of Tai Chi-based program can improve physical-function outcomes in stroke patients with impaired mobility. Performing Tai Chi for 6 months resulted in significant improvements in the flexor muscle strength of the involved knees. A previous study similarly found that older adults who practiced Tai Chi for 8 weeks showed significant improvements in their lower-limb muscle strength [35]. In contrast to normal walking, during Tai Chi stepping the knee joint always remains flexed with increased knee flexion angles and peak muscle activity [36]. Improvements in flexor muscle strength and mobility are critical for stroke survivors to be able to perform the ADL and avoid falls [37]. 

Along with the flexor muscle, the subjects in the Tai Chi group in the present study also showed significant improvements in their ambulation ability and the performance of the ADL, which would lead to significant improvements in the self-care dimensions of the QOL in this population. The typical feature of Tai Chi walking with weight transferal while stepping has been suggested as an effective mechanism for enhancing balance and mobility function [38]. 

However, no significant change in balance was found after performing Tai Chi for 6 months. Considering that stroke is typically a chronic disease, stroke survivors may require longer periods of Tai Chi training to achieve significant changes in balance. A systematic review [37] found that Tai Chi produced significant improvements in balance as assessed using the BBS in a random-effects model, which suggests that the effects of Tai Chi on balance would vary according to the characteristics of the involved population. 

The present study also found that stroke survivors who performed an adapted form of Tai Chi for 6 months showed significant improvements in cognitive function compared with those in the symptom management group. The baseline scores for K-MOCA and K-MMSE indicate that the participants in both groups had mild cognitive impairment [39], and those who performed Tai Chi showed significant improvements in these scores over 6 months. Tai Chi is a mind–body exercise that has been recognized as an effective tool for improving cognition by training subjects to focus on breathing and movement sequences and to increase self-awareness [40]. Recent meta-analyses have supported this hypothesis, by showing that Tai Chi exerts positive effects on global cognitive, executive, and memory functions in older adults with or without cognitive impairment [17,40]. 

The main limitation of this study was the smallness of the sample, which potentially made it statistically underpowered. A larger-scale randomized controlled study will be needed to draw more-definitive conclusions. Another limitation is the heterogeneity of the study population, including various rehabilitation stages. The participants of our study were a relatively young group, which consequently limits the generalizability of the study findings. Future studies should explicitly evaluate the benefits to stroke survivors in different age groups as well as at different stages of stroke recovery and with varying levels of functional ability.

## 5. Conclusions

The present Tai Chi-based stroke rehabilitation program was well received by stroke survivors with various levels of disability as assessed using the MRS. Compared with their control counterparts, the participants in the Tai Chi showed significant improvements in swallowing-related symptoms, ambulation ability, flexor muscle strength, and cognitive function, and consequently also in the self-care dimensions of the SS-QOL. This program could be applied to stroke survivors for managing their stroke-related symptoms and improving their QOL in community rehabilitation settings. Further studies should apply Tai Chi-based stroke rehabilitation programs to larger samples in order to explore the effects of such programs on symptom management and specific functional abilities over various durations of stroke rehabilitation.

## Figures and Tables

**Table 1:** Result from homogeneity of the general characteristics at baseline (*N* = 34).

Characteristics		Control (n = 16) n(%) or Mean ± SD	Tai Chi (n = 18) n(%) or Mean ± SD	*t/*χ^2^	*p*
Age, years		57.18 ± 10.65	58.72 ± 17.13	–0.91	0.367
Duration of stroke, months		10.94 ± 8.50	7.58 ± 5.98	1.32	0.200
Sex	Male	11 (68.8)	10 (55.6)	0.62	0.429
	Female	5 (31.3)	8 (44.4)		
Education	≤Primary school (grade 6th)	3 (18.8)	4 (22.2)	1.32	0.725
	Middle school (grade 7th~9th)	2 (12.5)	2 (11.1)		
	High school (grade 10th~12th)	8 (50.0)	6 (33.3)		
	≥College	3 (18.8)	6 (33.3)		
Marital status	Married	11 (68.8)	15 (83.3)	3.71	0.447
	Other	5 (31.2)	3 (16.7)		
Employment	Yes	4 (25.0)	3 (16.7)	0.36	0.549
	No	12 (75.0)	15 (83.3)		
Income, month	<US $2000	11 (68.8)	8 (47.1)	6.22	0.101
	US $2000–4000	4 (25.0)	3 (17.6)		
	>US $4000	1 ( 6.3)	6 (35.3)		
Diagnosis	Infarction	8 (50.0)	12 (66.7)	0.97	0.324
	Hemorrhage	8 (50.0)	6 (33.3)		
Activities of daily living	Independent	12 (75.0)	12 (66.7)	3.90	0.143
	Assisted/dependent	4 (25.0)	6 (33.3)		
Comorbidity *	Hypertension	6 (37.5)	11 (61.1)	1.89	0.169
	Cardiovascular disease	3 (18.8)	2 (11.1)	0.39	0.648
	Diabetes	6 (37.5)	5 (27.8)	0.37	0.545
	Neurological disease	8 (50.0)	10 (55.6)	0.11	0.746
	Other	4 (25.0)	6 (33.3)	1.72	0.340

* Multiple responses are possible.

**Table 2:** Results from homogeneity tests of the study variables at baseline (*N* = 34).

		Control (n = 16) N (%) or Mean ± SD	Tai Chi (n = 18) N (%) or Mean ± SD	*t*	*p*
Symptom cluster	Mobility	31.38 ± 9.49	28.33 ± 8.29	1.00	0.326
	Sensory	13.06 ± 3.51	11.78 ± 2.82	1.18	0.246
	Cognition	17.37 ± 5.89	14.61 ± 3.39	1.69	0.099
	Communication	20.94 ± 7.94	19.61 ± 5.21	0.58	0.565
	Mood	27.25 ± 8.70	22.33 ± 5.59	1.93	0.065
	Swallowing	5.81 ± 1.87	5.72 ± 1.71	0.15	0.884
Muscle strength	Flexor-I	125.43 ± 37.71	118.93 ± 37.39	0.50	0.618
	Extensor-I	54.30 ± 20.24	56.11 ± 20.07	–0.26	0.795
Physical function	BBS	48.75 ± 6.03	48.89 ± 6.64	–0.61	0.548
	TIS	16.31 ± 3.32	17.67 ± 3.61	–1.46	0.154
	FAC	3.88 ± 0.62	3.72 ± 0.67	–0.32	0.750
	MRS	1.69 ± 0.79	1.78 ± 0.65	–0.37	0.717
	K-MBI	87.88 ± 10.89	87.67 ± 9.93	0.06	0.954
Cognitive function	K-MOCA	24.00 ± 4.56	24.06 ± 3.54	–0.04	0.968
	K-MMSE	26.25 ± 4.09	26.61 ± 3.90	–0.26	0.794
SS-QOL	Fatigue	9.06 ± 3.21	10.28 ± 3.01	–1.14	0.263
	Family support	9.18 ± 2.95	10.72 ± 2.59	1.62	0.116
	Language ability	17.69 ± 5.31	18.17 ± 6.15	–0.24	0.811
	Movement ability	18.25 ± 5.67	19.06 ± 4.21	–0.47	0.639
	Emotion	15.75 ± 5.34	17.56 ± 4.37	–0.28	0.781
	Social role	13.94 ± 5.20	13.56 ± 4.13	0.24	0.813
	Personality	9.31 ± 4.24	9.67 ± 3.65	–0.26	0.795
	Thought processes	8.69 ± 3.50	9.22 ± 2.58	–0.51	0.613
	Self-care	13.88 ± 3.61	12.11 ± 2.81	1.60	0.119

Abbreviations: SD, standard deviation; Extensor-I, extensor muscle strength of involved leg; Flexor-I, flexor muscle strength of involved leg; BBS, Berg Balance Scale; TIS, Trunk Impairment Scale; FAC, Functional Ambulation Category; MRS, modified Rankin Scale; K-MBI, Korean version of the Modified Barthel Index; K-MOCA, Korean version of the Montreal Cognitive Assessment; K-MMSE, Korean version of the Mini Mental State Examination; SS-QOL, Stroke-Specific Quality of Life.

**Table 3:** Effects of the Tai Chi-based stroke rehabilitation program on symptom clusters (*N* = 34).

Outcome		Baseline	3 Months	6 Months		*F*	*p*
Mobility	Tai Chi	28.33 ± 8.29	26.39 ± 8.08	26.22 ± 7.87	T	5.72	0.011
	Control	31.38 ± 9.49	28.75 ± 8.19	30.13 ± 7.75	G	1.29	0.264
					T × G	0.61	0.504
Sensory	Tai Chi	11.78 ± 2.82	11.28 ± 2.52	10.56 ± 2.25	T	2.98	0.058
	Control	13.06 ± 3.51	12.88 ± 3.03	12.31 ± 2.98	G	3.19	0.083
					T × G	0.17	0.783
Cognition	Tai Chi	14.61 ± 3.39	14.05 ± 3.81	12.22 ± 2.83	T	8.35	0.001
	Control	17.37 ± 5.89	15.87 ± 5.50	15.31 ± 4.22	G	3.54	0.069
					T × G	0.73	0.482
Communication	Tai Chi	19.61 ± 5.21	17.83 ± 4.37	16.50 ± 4.23	T	4.57	0.014
	Control	20.94 ± 7.94	21.31 ± 7.91	20.19 ± 6.49	G	2.07	0.016
					T × G	2.04	0.138
Mood	Tai Chi	22.33 ± 5.59	21.94 ± 7.23	20.11 ± 5.49	T	2.60	0.097
	Control	27.25 ± 8.70	25.69 ± 8.87	25.38 ± 8.42	G	3.95	0.056
					T × G	0.39	0.624
Swallowing	Tai Chi	5.72 ± 1.71	5.06 ± 1.39	5.39 ± 1.42	T	1.61	0.206
	Control	5.81 ± 1.87	7.31 ± 2.82	6.19 ± 1.64	G	3.50	0.070
					T × G	8.96	0.001

**Table 4:** Effects of the Tai Chi-based stroke rehabilitation program on muscle strength, physical function, and cognitive function (*N* = 34).

Outcome		Baseline	3 Months	6 Months		*F*	*p*
Flexor-I	Tai Chi	118.93 ± 37.39	129.33 ± 36.17	137.70 ± 33.32	T	0.94	0.377
	Control	125.43 ± 37.71	119.51 ± 38.07	116.00 ± 31.56	G	0.51	0.479
					T × G	8.61	0.002
Extensor-I	Tai Chi	56.12 ± 20.08	59.84 ± 18.57	59.80 ± 20.73	T	1.37	0.260
	Control	54.31 ± 20.24	58.97 ± 23.02	55.92 ± 19.77	G	0.12	0.733
					T × G	0.18	0.782
BBS	Tai Chi	48.89 ± 6.64	51.33 ± 5.77	53.59 ± 7.64	T	35.31	<0.001
	Control	48.75 ± 6.03	50.06 ± 5.93	52.25 ± 3.61	G	0.18	0.677
					T × G	0.85	0.434
TIS	Tai Chi	17.67 ± 3.61	19.33 ± 3.18	20.72 ± 2.89	T	44.18	<0.001
	Control	16.31 ± 3.32	18.50 ± 3.10	19.25 ± 3.34	G	1.35	0.255
					T × G	0.55	0.556
FAC	Tai Chi	3.72 ± 0.67	3.94 ± 0.73	4.22 ± 0.55	T	7.03	0.002
	Control	3.88 ± 0.62	3.81 ± 0.54	3.88 ± 0.62	G	0.30	0.590
					T × G	6.78	0.002
MRS	Tai Chi	1.78 ± 0.65	1.67 ± 0.59	1.56 ± 0.78	T	0.25	0.782
	Control	1.69 ± 0.79	1.81 ± 0.75	1.81 ± 0.83	G	0.20	0.657
					T × G	2.12	0.129
K-MBI	Tai Chi	87.67 ± 9.93	89.50 ± 8.35	92.78 ± 7.95	T	14.21	<0.001
	Control	87.88 ± 10.89	86.13 ± 13.09	89.88 ± 10.61	G	0.36	0.555
					T × G	3.23	0.046
K-MOCA	Tai Chi	24.06 ± 3.54	25.33 ± 4.09	26.67 ± 3.25	T	3.62	0.044
	Control	24.00 ± 4.56	23.69 ± 6.31	23.56 ± 5.33	G	1.14	0.294
					T × G	7.09	0.004
K-MMSE	Tai Chi	26.61 ± 3.90	27.11 ± 3.38	28.06 ± 2.98	T	0.98	0.381
	Control	26.25 ± 4.09	25.69 ± 4.60	25.63 ± 3.76	G	1.29	0.265
					T × G	4.33	0.017

Abbreviations: SD, standard deviation; Extensor-I, extensor muscle strength of involved leg; Flexor-I, flexor muscle strength of involved leg; BBS, Berg Balance Scale; TIS, Trunk Impairment Scale; FAC, Functional Ambulation Category; MRS, modified Rankin Scale; K-MBI, Korean version of the Modified Barthel Index; K-MOCA, Korean version of the Montreal Cognitive Assessment; K-MMSE, Korean version of the Mini Mental State Examination; SS-QOL, Stroke-Specific Quality of Life.

**Table 5:** Effects of the Tai Chi-based stroke rehabilitation program on Stroke-specific Qaulity of Life (SS-QOL) (*N* = 34).

Outcome		Baseline	3 Months	6 Months		*F*	*p*
Energy	Tai Chi	10.28 ± 3.01	9.56 ± 2.85	9.00 ± 2.28	T	2.11	0.130
	Control	9.06 ± 3.21	10.00 ± 3.08	9.00 ± 2.92	G	0.09	0.771
					T × G	2.19	0.120
Family roles	Tai Chi	10.72 ± 2.59	10.44 ± 3.24	10.22 ± 3.42	T	0.03	0.972
	Control	9.19 ± 2.95	9.56 ± 3.10	9.88 ± 3.05	G	0.93	0.341
					T × G	1.16	0.321
Language	Tai Chi	18.17 ± 6.15	19.72 ± 3.64	18.67 ± 4.46	T	0.54	0.585
	Control	17.69 ± 5.31	17.25 ± 5.78	16.69 ± 4.67	G	1.24	0.274
					T × G	0.85	0.418
Mobility	Tai Chi	19.06 ± 4.21	21.89 ± 4.56	20.72 ± 4.97	T	2.80	0.079
	Control	18.25 ± 5.67	18.81 ± 6.31	17.38 ± 6.41	G	2.19	0.149
					T × G	1.73	0.191
Mood	Tai Chi	16.22 ± 4.51	17.56 ± 4.37	17.78 ± 4.70	T	0.54	0.583
	Control	15.75 ± 5.34	15.69 ± 3.63	14.69 ± 3.94	G	1.78	0.191
					T × G	2.27	0.123
Social roles	Tai Chi	13.56 ± 4.13	14.28 ± 4.04	16.11 ± 5.09	T	3.01	0.056
	Control	13.94 ± 5.20	14.63 ± 5.51	15.19 ± 4.13	G	0.002	0.962
					T × G	0.45	0.617
Personality	Tai Chi	9.67 ± 3.65	9.89 ± 3.01	11.17 ± 3.24	T	5.97	0.004
	Control	9.31 ± 4.24	9.69 ± 3.96	10.38 ± 3.38	G	0.15	0.698
					T × G	0.31	0.733
Thinking	Tai Chi	9.22 ± 2.58	10.44 ± 2.87	11.33 ± 2.74	T	7.93	0.001
	Control	8.69 ± 3.50	9.38 ± 2.47	9.06 ± 2.64	G	2.10	0.157
					T × G	3.70	0.030
Self-care	Tai Chi	12.11 ± 2.81	12.56 ± 3.15	18.78 ± 5.06	T	52.00	<0.001
	Control	13.88 ± 3.61	12.06 ± 3.62	16.00 ± 3.93	G	0.20	0.661
					T × G	8.82	0.001

## Data Availability

The datasets used and/or analyzed during the current study are available from the corresponding author (M.P.) on reasonable request.
